# Managers perspectives on the implementation of the Prevention of Mother-to-Child Transmission policy in South Africa

**DOI:** 10.4102/jphia.v17i1.1551

**Published:** 2026-04-09

**Authors:** Hermina Dyeshana, Sibusiso Zuma

**Affiliations:** 1 Department of Health Studies, Faculty of Human Sciences, University of South Africa, Pretoria, South Africa

**Keywords:** perceptions, prevention, mother-to-child transmission, elimination, non-adherence, interventions, strategies, guidelines

## Abstract

**Background:**

Despite significant progress in South Africa in reducing mother-to-child transmission of human immunodeficiency virus (HIV) infection, challenges remain. This is evident by the incidence and prevalence of HIV in pregnancy and transmission during breastfeeding. This study is aimed at ascertaining the perceptions of prevention of mother-to-child transmission (PMTCT) managers regarding the challenges experienced during the implementation of the PMTCT policy at public health service delivery platforms across all provinces of South Africa.

**Aim:**

To examine the perceptions and views of PMTCT managers regarding the challenges experienced during implementation of the policy.

**Setting:**

The study was conducted in all nine provinces of South Africa.

**Methods:**

An exploratory-qualitative descriptive research design was utilised. The study population was the PMTCT managers. Data were collected from a sample of 11 PMTCT managers, seven at the provincial level, three at the district level, and one at the national level, using in-depth semi-structured interviews from April 2021 to July 2022. The participants were purposefully selected based on their expertise and experiences related to the study topic.

**Results:**

The participants shared the views, experiences and challenges encountered during the PMTCT policy implementation. Four themes were generated: policy design and change management, system and service delivery constraints, provider-level determinants and client-level determinants. It is imperative that recommendations shared be implemented to support the full implementation of the PMTCT policy, enabling the country to achieve the elimination of mother-to-child transmission by 2030 and an HIV-free generation.

**Conclusion:**

The article confirmed that the Prevention of Mother-to-Child Transmission policy is partially implemented in South Africa which may derail the country to achieve eMTCT by 2030.

**Contribution:**

The article propose guidelines towards full implementation of the Prevention of Mother-to-Child Transmission policy across all service delivery platforms across South Africa.

## Introduction

Significant progress has been made since the 2001 inception of the national Prevention of Mother-to-Child Transmission (PMTCT) programme in South Africa (SA). However, bottlenecks and challenges continue to persist that hamper the elimination of mother-to-child transmission (eMTCT) of human immunodeficiency virus (HIV).

There is a noticeable prevalence and new incidences of mother-to-child transmission (MTCT) of HIV. This is attributed to infection during pregnancy or breastfeeding,^1^ with this mode of transmission accounting for 50% of paediatric HIV infection transmission in MTCT of HIV worldwide.^2^ A similar concern was raised about women and infants acquiring HIV, despite universal test and treat (UTT)-based PMTCT in place, because of suboptimal post-natal adherence to maternal antiretroviral therapy (ART). These findings emphasise that the current application of the PMTCT policy will not be sufficient to eliminate MTCT, particularly post-natal MTCT. Urgent action is therefore needed to evaluate and implement complementary biomedical preventive strategies to eliminate postnatal MTCT.^3^

South Africa is among the countries that adopted interventions of the Global Plan in 2011 towards the elimination of new HIV infections among children and keeping their mothers alive by 2015.^4^ In 2015, the SA Ministry of Health PMTCT policy introduced lifelong triple ART for all HIV-positive pregnant and lactating women (PMTCT Option B+) and 3-monthly HIV testing of HIV-negative pregnant and lactating women, including birth polymerase chain reaction (PCR) testing at 10 weeks. The birth PCR test provides an opportunity for the early identification of babies who have acquired HIV in utero and links them to HIV care and treatment as early as possible.^5^

The SA Ministry of Health rolled out PMTCT policy guidelines and adopted strategies, including the District Implementation Plan (DIP) process, mandated by the United Nations Programme on HIV/AIDS (UNAIDS), to ensure the mission of an acquired immunodeficiency syndrome (AIDS)-free generation by 2030.^6^ Post-review, SA, as a developing country, is committed to decreasing MTCT rates and new HIV infections in children as it moves towards the goal of an HIV-free generation by utilising the ‘Last Mile Plan’. The rationale for the Last Mile Plan is that, despite significant progress since 2008, bottlenecks and challenges remain that hamper eMTCT. The goal of the ‘Last Mile Plan’ is a further reduction from the then-current baseline of MTCT of HIV and new HIV infections in children, with an end goal of eMTCT by 2030.^7^

Prevention of Mother-to-Child Transmission policy changes rollout and the action framework implemented in the country over the last 6 years facilitated and yielded a reduction in the vertical transmission of HIV from mothers to babies, including post-partum transmission risks for gestation of around 10 weeks, from 3.5% in 2009 to 1.1% in 2016.^8^

The annual 2018–2019 National Department of Health (NDoH) report displayed results showing a reduction in the MTCT rate of around 10 weeks, from 1.3% in the 2016/2017 Financial Year (FY) to 0.9% in 2017/2018 FY and 0.74% at the end of the 2018/2019 FY.^9^

A sharp decline in the MTCT rate of around 10 weeks was also reported in 2020 for respective provinces, namely the Eastern Cape (EC) (1.0%), the Free State (FS) (0.6%), Gauteng (GP) (0.7%), KwaZulu-Natal (KZN) (0.6%), Limpopo (LP) (0.7%), Mpumalanga (MP) (0.9%), the Northern Cape (NC) (1.4%), the North West (NW) (0.9%) and the Western Cape (WC) (0.3%).^10,11^ A further decline in the MTCT rates of around 10 weeks was reported in the District Health Information system repository (DHIS 2021) for the six provinces, namely EC (0.7%), FS (0.5%), KZN (0.4%), MP (0.6%), NC (1.0%) and NW (0.7%). The MTCT rates remained static in three provinces, namely, GP (0.7%), LP (0.7%) and WC (0.3%).

It has been highlighted that, despite the country’s PMTCT policy guidelines and strategies being among the best in Africa and demonstrating a decrease in the national risk of early MTCT, implementation gaps persist, and there is a noticeable prevalence and new incidence of MTCT.^13^ This is attributed to women being infected during pregnancy or transmission during breastfeeding.^14^ The literature review identified several studies that revealed gaps in PMTCT policy implementation that could hinder the country’s efforts to achieve eMTCT by 2030.^15,16,17,18,19^

Few studies have examined why there are gaps in the implementation of the PMTCT policy or why it is not fully implemented in-service delivery platforms in SA, prompting the researcher to explore the rationale for these gaps.

## Research methodology and design

Qualitative research with an exploratory-descriptive design was utilised to explore and describe the experiences, perceptions and views of PMTCT managers to establish why PMTCT policies are not fully implemented at public service delivery platforms rendering PMTCT services in SA, as well as describing obstacles derailing full implementation of the PMTCT policy. Prevention of Mother-to-Child Transmission managers were interviewed using an in-depth semi-structured interview technique until data saturation was reached.

### Setting, sampling and sample size

Prevention of mother-to-child transmission managers were purposively selected based on their expertise and experience with the study topic. Moreover, these participants have undergone training and have formal education to manage the PMTCT programme.

A sample of seven provincial PMTCT managers representing EC, FS, LP, KZN, MP, NC and NW provinces was interviewed. Prevention of Mother-to-Child Transmission managers of three districts were interviewed, with two representing WC because the PMTCT manager post was phased out during their restructuring. This province is practising a district-based functional structure, whereby each district has a PMTCT manager. One of the two district PMTCT managers represented the metro district, and the other represented the rural district. One district manager representing the Tshwane district in the GP district, because of the unavailability of the provincial PMTCT manager and one national PMTCT manager were interviewed, which ensured transferability representation of the nine provinces of SA where PMTCT services are rendered, inclusive of the national level to share an overall country view.

The participants (PMTCT managers) were approached by the researcher and invited to participate in the study to share their perceptions, views and experiences regarding the topic, as they are the custodians of the programme in their respective districts and hold decision-making authority.

All 11 participants were female, professional nurses and qualified midwives. The participants had in-depth experience ranging from 4 years to more than 15 years in all obstetric and maternity service delivery platforms, namely antenatal care, all areas in the maternity wards, labour wards, theatre, obstetrics, and gynaecology, as well as post-natal wards. The professional grades of the participants ranged from Director level (*n* = 1), to Deputy Directors (*n* = 8) and Assistant Directors (*n* = 2).

### Data collection

Data collection was conducted from 19 April 2021 to 05 July 2022. The researcher obtained the PMTCT managers’ consent to participate using Microsoft Teams and/or Zoom (virtual interviews). In-depth, audiotaped interview sessions lasting between 45 min and 60 min were conducted, accompanied by field notes taken to maintain reflexivity, supplement and include what was elaborated by the participant upon a particular question being asked the same way to all participants. Data saturation was reached when similar answers were given, and no new concepts or information emerged during the interview sessions. Interviews were conducted in English.

An interview guide with probing questions was used, with the following questions being asked: ‘What are the perceptions of PMTCT managers regarding the implementation of PMTCT when delivering care to pregnant women?’, ‘How did PMTCT managers experience the implementation of the PMTCT policy when delivering care to pregnant women?’ and ‘What are the challenges associated with the implementation of the PMTCT policy in the identified provinces?’.

Each interview was audiotaped and labelled, using a unique identification number, rather than the names of the provinces. A transcriber was employed to listen to the audiotapes, extract the information shared and document it verbatim in sequence after the researcher had conducted the interviews, using exact codes for each specified date. The researcher checked the transcripts and sent them to the respective participants for verification and accuracy.

### Data analysis

ATLAS.ti version 8 software was used for the data analysis process, using a reflexive thematic analysis systematic procedure methodology comprising six steps.^20^ The first step included familiarisation with the collected data, whereby interview transcripts were transcribed verbatim to ensure trustworthiness and accuracy, followed by the generation of initial codes, which involved systematically working through the data to identify significant features. In this step, the interview responses were examined in detail to derive and assign codes to the meaningful segments of the text relevant to the research question. Descriptive codes were used to summarise the participants’ responses.

The third step involved searching for themes in which similar codes were grouped to identify a theme, followed by reviewing the themes to assess whether the identified codes fit the respective themes and to evaluate the themes represented in the participants’ responses. Themes that did not correspond to the identified codes were discarded.

The next step was to define and name the themes, produce a report assigning brief definitions to the refined themes and describe how the themes related to the research questions. Concise, reflective and descriptive names were assigned to the themes, with each theme being supported by direct participant quotations and a detailed description to ensure clarity.

The final step in the thematic analysis was to write the report, in which the refined themes were translated into a transparent narrative. A report was written on the findings to provide a clear rationale on how the themes were developed, supported by direct participant quotations, to demonstrate key points and communicate the research findings to the respective audience. The themes were described and interpreted in a way that aligned with and answered the research question.

A co-coder was sought to support the researcher with coding and applying inductive and deductive approaches. Researcher bias and personal assumptions were acknowledged and bracketed, which facilitated the resolution of disagreements through discussions with the co-coder and highlighted differences in how narratives were interpreted and presented. An agreement on transparency was reached with the co-coder, with the understanding that differences in interpretation and data presentation would be resolved through discussion until consensus was achieved.

Finalisation of the themes process started by understanding the meaning of the data and identifying themes and subthemes from the collected data by highlighting ‘significant statements’ made, narrowing them into themes to obtain detailed descriptions of what and how PMTCT managers experienced PMTCT implementation, as well as why PMTCT policies were not being fully implemented, which was followed by verification, completeness coding and the data being captured in an Excel spreadsheet. The data were then transferred to ATLAS.ti version 8 for analysis, with the subthemes organised thematically. The content of each theme was summarised to draw meaningful conclusions through interpretation.

Researcher bias and personal assumptions were acknowledged to ensure trustworthiness by enhancing credibility and acceptance of the findings and reflexivity, which was assured by the credentials and professional background of the researcher who is a clinician and a nurse by profession, with additional nursing qualifications, namely Master of Science in Nursing, Higher Diploma in Nursing Management, Bachelor of Nursing Science in Education, Diploma in Child Nursing, Higher Diploma in Community Health Nursing, Diploma in Psychiatric Nursing, Diploma in Midwifery and Diploma in General Nursing Science, with additional qualifications as an Occupational Health and Safety Specialist, an accredited Outcomes-Based Assessor, an accredited Outcomes-Based Moderator and an HIV Practitioner who worked closely with the provincial PMTCT managers as a colleague whereby strong relationships were formed from 2009 to 2016. The researcher also served as a provincial HIV Prevention Manager, responsible for programme management of PMTCT and other HIV prevention programmes.

During the research interviews, there was prolonged engagement, with sufficient time spent with PMTCT managers from the time of their invitation to participate in the study and throughout the interviews. The interviews lasted 45 min – 60 min per participant, collecting data and creating rapport and an atmosphere of trust.

Bias and personal assumptions were acknowledged by the researcher employing strategies to ensure that the study was trustworthy, dependable and of high quality, enhancing credibility, acceptance of findings and reflexivity. The criteria of trustworthiness are put forward as credibility, transferability, dependability, integrity and applicability,^21^ which were used to establish the trustworthiness of the study. The researcher purposively selected PMTCT managers who were experts in the programme in which the study was conducted. The experiences they shared and documented in the form of data are credible, as they are knowledgeable about the PMTCT policy and have identified implementation gaps. Therefore, they shared factual, credible information (findings), which was documented.

The approaches used in the study to ensure credibility are described as follows.

*Spending time with the participants* (prolonged engagement)^22^: The researcher invested sufficient time collecting data, spending time with the participants, creating rapport and an atmosphere of trust. This made the participants comfortable enough to answer the questions posed to them.

*Member-checking* is described as a critical technique used to establish credibility, whereby participants participate in reviewing data and findings to ensure that their perspectives were captured correctly.^21^ The researcher shared the interview scripts used during interviews with PMTCT managers for their review and verification of the information captured, to check the accuracy of the facts, and to validate the summary draft of the research report.

All participants received written narrative reports of the transcribed interviews, highlighting identified gaps in their respective provinces and including recommendations from the researcher to support efforts to mitigate these gaps. Receipt of the reports was acknowledged by all participants with appreciation, and remedial action processes initiated in the respective provinces were shared with the researcher. Some participants reported improved performance where gaps had been addressed.

Reflexivity is described as awareness that the researcher, as an individual, brings to the inquiry – a unique background, a set of values, and a social and professional identity of delimiting subjectivity – that can affect the research process by practising self-bracketing, self-interrogation and reflection.^21,22^ The researcher avoided compromising meaningful, trustworthy data by practising self-awareness as an individual, as well as self-bracketing to avoid producing a biased report.

**Confirmability:** The interviews with PMTCT managers were transcribed, and the transcripts were sent to the respective managers for verification and validation to ensure that the transcribed material accurately reflected their views.

**Dependability:** Prevention of Mother-to-Child Transmission managers, as programme managers, have programme management oversight and are accountable for quality improvement. Whether they are interviewed by other researchers or the study is repeated or replicated in the same or a similar context, the findings would be the same. Their experiences and perceptions regarding implementation gaps and the rationale will not change, as they are their own and will remain the same; hence, the data collected over time will be the same. The information shared by the PMTCT managers was credible and reliable and therefore reliable for informing decision-making processes for guideline development.

**Transferability:** The PMTCT managers’ views and perceptions were detailed to the extent that the researcher was able to gather sufficient information. Transferability was also enhanced by a clear description of the research process and all methodological aspects.

Integrity is described as ongoing self-reflection and self-scrutiny to ensure that interpretations of data are valid and grounded in the data.^22^ The researcher maintained and demonstrated professionalism by engaging in ongoing self-reflection and self-critique to ensure that interpretations of the data were valid and grounded. Data analysis took place from 02 November 2021 to 05 July 2022. The reviewed literature was used to support the findings.

### Ethical considerations

Basic ethical principles of autonomy, justice, beneficence, anonymity and non-maleficence for the participants, researcher and institutions were observed and addressed through approval sought from the Department of Health (DoH). Authorisations were obtained, which protected the rights of the public health facilities where information (data) was collected, as well as those of the participants who consented to participate in the study.

Participation was voluntary. All the participants were assured of confidentiality and anonymity. Informed written consent was obtained after the participants had read and understood the information letter issued to them. Confidentiality was maintained by replacing participants’ real names with provincial code names and numbers. Only the researcher and the transcribers could match them.

The study was conducted in partial fulfilment of the requirements for a PhD in Public Health at the University of South Africa. All procedures related to the study were performed in accordance with ethics guidelines approved by the Ethics Committee of the University of South Africa. Health Higher Degrees Ethics Review Committee approved the research study. Ethical clearance (certificate permit number: HSHCD/1007/2020) was obtained.

The transcribed documents were kept in a filing cabinet – locked away for confidentiality purposes – until such time that the research report was completed and submitted as a thesis, following which it was destroyed and disposed of accordingly (shredded) to safeguard the participants’ confidential information. It was agreed that the findings and recommendations would be made available to the relevant authorities, and a copy of the thesis would be kept in the university’s online research repository housed within the library.

## Results

In total, 11 participants, aged 35–60 years, participated in the study. Two (18.2%) had been managing the PMTCT programme in their respective areas for 5 years. Three (27.3%) from 6–9 years, five (45.5%) for 10–15 years, and one (9.1%) had been with the programme for more than 15 years.

All 11 participants were females, professional nurses and qualified midwives with the highest educational qualification of either a diploma, degree or master’s degree. Four themes and 20 subthemes emerged during data analysis. A narrative account of the themes and subthemes, supported by direct quotations from participants, is presented in Table 1.

**TABLE 1:** Themes and subthemes emerging from the collected data.

Themes	Subthemes
1. Policy design and change management	1.1. Policy obstacles 1.2. Frequent guideline change 1.3. Communication pathways
2. System and service delivery constraints	2.1. Staffing levels 2.2. Lack of resources 2.3. Referral systems 2.4. Overcrowding 2.5. Reliance on support partners 2.6. Poor integration of services 2.7. Mother-baby pair management
3. Provider-level determinants	3.1. Training quality challenges 3.2. Guideline literacy 3.3. Documentation and data quality 3.4. Health care provider challenges 3.5. Management support
4. Client-level determinants	4.1. Late ANC booking 4.2. ART adherence 4.3. Denial of HIV status by pregnant women 4.4. Stigma 4.5. Lost to follow-up

ANC, antenatal care; ART, antiretroviral therapy; HIV, human immunodeficiency virus.

### Theme 1: Policy design and change management

Three subthemes emerged under theme 1, and the supporting narratives are presented verbatim as follows.

#### Subtheme 1.1: Policy obstacles

Eight provincial PMTCT managers perceived that PMTCT policy was partially implemented in their respective provinces, as evidenced by their statements. Two of the provincial managers were of the impression that the policy is fully implemented in their respective provinces, but both acknowledged gaps as challenges. The following are examples of verbatim responses from the participants:

‘I would say, according to my perceptions, the policy, or the guidelines, are not implemented properly, as they should be.’ (P7, female, manager)

‘My perception of the implementation of the PMTCT programme is that the programme is implemented in the province, at all our health facilities. It has got its challenges that some of them are client-related, some of them are system-related, and some are based on how the service providers are managing the programme, and some of them even emanate from the policy guideline itself.’ (P8, female, manager)

#### Subtheme 1.2: Frequent guideline changes

The findings revealed that frequent policy and guideline changes are one of the main issues hindering quality care, with unwarranted negative outcomes derailing the implementation of the PMTCT policy, evidenced by the following narrated statements:

‘Guidelines keep on changing, and you know what, I remember when we had to implement this thing of birth PCR. I remember it was during the training when we told them. They said that you keep giving us more work on top of the very same people, and you don’t even give us more resources. They said that you know, we don’t even have time to do other things. It was a birth PCR. Now it is VL drawing bloods for VL monitoring during delivery, and remember delivery viral loads has to be done also in the labour ward. They then do not do both interventions, leading to non-compliance and the policy not being implemented fully, interventions are not being done.’ (P2, female, manager)

‘Another issue is that this policy changes anytime. It’s so dynamic. I am told it will change soon, so it changes now and again. Each time it changes, people just get overwhelmed because it’s when they just start to enjoy and to understand how they supposed to do things, then policy changes to something new, and then as it changes, they get overwhelmed, they are just shocked and they get so confused and each time there’s policy changes, there will be a decline in figures in terms of the stats, the performance.’ (P6, female, manager)

#### Subtheme 1.3: Communication pathways

Some of the participants raised a concern of PMTCT managers being left behind and only Regional Training Centre (RTC) managers being invited for training, which was viewed as a lack of communication on the part of the NDoH, regarding changes made as to which cadre of staff needed to cascade training at the provincial level.

As with previous PMTCT policy changes, PMTCT managers were the only ones trained with the expectation that, upon return to the province, they would cascade training. During the dissemination of the 2019 policy changes by the NDoH, only invited RTC managers were trained. Upon return to the province, they did not train any personnel regarding PMTCT policy changes. It was then expected of PMTCT to conduct training, yet they were not trained. The issue was raised and was reported to have resulted in role confusion between RTC managers and PMTCT managers. The narrative raised by two participants is presented verbatim as follows:

‘The confusion of who was expected to do cascade training at the provincial level on the 2019 guideline amendments was confusing. As with previous guideline training, PMTCT managers were the ones trained to further cascade provincial PMTCT training; however, they were left behind and were not invited for training. When PMTCT managers requested clarity from NDoH regarding who was expected to conduct provincial PMTCT cascade training, seeing that they were not trained, no answers were given to them.’ (P7, female, manager)

‘The changing guideline, remember, the National Department of Health would invite … like in this instance, they invited the RTC managers to go and attend training; so, after the training, then coming back to the province, they were to start with training the healthcare providers. That was in 2020, because the training happened around October and November. That PMTCT guideline changes training never happened.’ (P8, female, manager)

### Theme 2: System and service delivery constraints

It was found in this study that system and service delivery were negatively impacted by staffing issues, mainly because of staffing levels or inadequate staffing, staff attrition and the rotation of personnel, supply chain inadequacy, referral systems, overcrowding, reliance on partners, as well as the integration of services and mother-baby pair management. All the participants highlighted the challenges grouped into seven subthemes that emerged.

#### Subtheme 2.1: Staffing levels

The participants raised concerns of staff attrition and rotation, which were attributed to various factors. Examples of participants’ views are presented as follows:

‘Some of the reasons for gaps we have regarding full implementation are because of staff attritions and non-continuation of training.’ (P5, female, manager)

‘We’re having a high staff turnover in our province, because people will be looking for greener pastures, so they will leave, which results in clinicians being overwhelmed.’ (P7, female, manager)

Seven participants reported that clinicians are overwhelmed, citing various scenarios that contribute to overwork, including staff shortages, attrition and rotation. An example of the views shared is as follows:

‘Clinicians are overwhelmed. They have got quite a few clients waiting for them outside. So, for them to be reaching out to a guideline and to read through, they feel it is a time-waster. But they do not realise that they may end up not managing the client properly, because of not being well-informed. The one major area is overcrowding that is experienced in our facilities, and overcrowding, a large number of clients meant to be managed by one clinician.’ (P3, female, manager)

#### Subtheme 2.2: Lack of resources

The majority of participants highlighted a lack of and poor allocation of resources in some provinces, which was viewed as derailing the implementation of necessary interventions to achieve programme outcomes. Lack of resources included shortages and inadequate staffing, a lack of managerial support, and the unavailability of ART drug regimens. Examples of participants’ views on the issue are presented in verbatim as follows:

‘I was frustrated. When I came back from the support visit, from one of … the CHC. The professional nurse there, working as the Acting Operational Manager, was alone, alone, alone! When we got there, we could not do a thing, as she was so exhausted, because she had worked a 12-hour shift and then a night shift. She was coming back later for the night shift. She did not even go home; she was still there for another 12-hour shift of the third day.’ (P7, female, manager)

‘There’s no adequate staffing, but on top of that, what we emphasise to clinicians to attend to the client’s needs 100%, even if we say, we don’t have resources or enough staff.’ (P2, female, manager)

Nevirapine (NVP) was unavailable at some facilities because of a failure by one of the districts’ medical depots to procure adequate NVP supplies, resulting in a stockout. The narrative provides evidence that facilities were not able to give HIV-exposed infants prophylaxis to reduce their chances of MTCT. This led to facilities being viewed as non-compliant to the PMTCT policy, resulting in poor programme outcomes, as those infants had the potential of seroconverting:

‘System challenge, it is like I have mentioned that, sometimes you find that the availability of some of the drugs is based on the changes in the regimen, which also has an impact on us being able to immediately implement the new changes that are in the guidelines. We started very well when the guideline was initially introduced. We did have the necessary stock, but then, with time, we were not having the right – how can I put it? We did not have the Nevirapine syrup. The depot had inadequate stock to cater for all our infants. The … district was unable to give the Nevirapine suspension to those HIV-exposed infants.’ (P8, female, manager)

#### Subtheme 2.3: Referral system

Non-functional referral systems are viewed as multifactorial challenges affecting referral systems between facilities, districts, provinces and inter-country. They are perceived as major gaps that lead to poor client management, as indicated in the following quotes:

‘Non-functional referral systems between facilities delivering ante-natal or post-natal services, in clinics or hospitals around provinces, inter-provincial referrals, and inter-country referrals create a huge gap in client management as our communities are mobile. The systems should be enabling a proper functional referral system to give the clinician direction and information regarding clients being referred for post-natal care, because we are treating communities who are very much mobile.’ (P10, female, manager)

‘You know, clients move provinces; they move around countries as well. When this client comes to any point in South Africa, if there were a proper referral system, and not dependent on the client, who should be open about their status to say, I was in Clinic A, and I was tested for viral load, and I didn’t get the results. Her results can be accessible wherever she is if she has the barcode. Clinicians have their own part to play and even our clients.’ (P3, female, manager)

#### Subtheme 2.4: Overcrowded facilities

These facilities are perceived as unsuitable environments, creating obstacles for clinicians to implement necessary interventions and adhere to PMTCT guidelines. This was verbalised as follows:

‘The one major area with overcrowding experienced in our facilities is that one clinician has to manage the entire facility workload, and they are overwhelmed as they have several clients waiting for them outside the consulting rooms. Remember, the issue of overcrowding is attributed to the number of staff members versus the number of clients that are visiting the facility. I mean, it has been clear that with an increasing number of clients accessing facilities, there is no equivalent increase in the number of clinicians allocated to those facilities.’ (P3, female, manager)

‘The systemic issues that I am talking about are the issue of the number of personnel in a facility based on the population that they are serving. You’ll find out that the other facilities, like those in urban areas, have more staff. Then you’ll find out, in areas where it’s rural, or these klein plekkies, or smallanyana towns, you’ll find out, it’s only one professional nurse serving a lot of clients alone, who will say I was alone in the facility and the queue was long, everybody was waiting for me.’ (P3, female, manager)

#### Subtheme 2.5: Reliance on support partners

Dependency and reliance on funded partners’ support were found to be a challenge in some facilities and provinces, whereby accountability and responsibility of the departmental data capturers was shifted to the partners’ data capturers. Is there a lack of continuity, or is there no sustainability plan in place for the government to ensure that the intervention and/or data capture continues in the absence of donor and/or implementing partners’ support? Verbatim responses are presented as follows:

‘Provincial monitoring and evaluation personnel do not have ownership of exercising their responsibility, but are dependent on the support of the supporting partner for the monitoring of the quality improvement plan, which, in essence, represents reliance on partners.’ (P4, female, manager)

‘Funded partners assist us. We would then get these data capturers that are allocated to our sites to do the capturing for us. The one weakness of our facilities is that once the funded partner’s data capturer has been allocated there, they would take out the departmental government capturers from that room and place them elsewhere. In the absence of funded partners due to unforeseen circumstances, no capturing takes place, which tells me that the coordination and the supervision are a weakness and a challenge at those facilities, which contributed to the missed opportunity.’ (P9b, female, manager)

#### Subtheme 2.6: Poor integration of services

Three provinces attributed poor service integration to inadequate programme management, resulting in partial implementation of the PMTCT policy in their provinces. Participants in this study indicate that poor service integration contributes to partial policy implementation, as interventions are not implemented as intended, resulting in missed opportunities for client care:

‘The one other area that I have seen as being a challenge is failure to integrate services for child health. In EPI, we focus on immunising; somebody is doing PMTCT there, somebody is attending to IMCI. Ideally, all three must be in one room, and that would actually encourage clinicians to check, because they will be checking for everything that needs to be checked there for a baby, even manage appropriately so.’ (P3, female, manager)

‘If negative children who have been aligned for the testing, the follow-up of HIV-exposed children with the EPI, so that when they come for immunisation, they can be picked up. But we still are seeing a gap where, you know, our coverage of immunisation is good, but our follow-up for HIV-exposed infants is not good at all, meaning that there is a gap. I do not think integration is a problem. I think if we were using it, we could have good benefits from it.’ (P10, female, manager)

#### Subtheme 2.7: Mother-baby pair management

The participants highlighted a gap whereby mothers and babies are not being attended to as a pair at facilities because of poor programme management. Verbatim responses on the narrative are presented as follows:

‘With that one, we are still lagging. In terms of the mother and baby pairs. Remember, we are faced with a situation of teenage pregnancy. You will only find the mother being able to take the baby to the facility six days post-natal. Otherwise, moving forward, you find that the baby will be taken by either a grandmother or the guardian, because the mother has gone back to school.’ (P1, female, manager)

‘When the mother is coming for the post-natal visit, three to six days after delivery, you’ll find out when you check the file that the mother and baby pair were there, but only the observation of the mother was done. They did not check the baby. Now, you ask yourself, what type of post-natal care is this one? When you are not going to check both clients, yet we are supposed to be checking this pair, from head to toe, both of them, and also check on our checklist as to what is supposed to be done at this interval, but we’re not doing that.’ (P7, female, manager)

### Theme 3: Provider-level determinants

Training quality, guideline literacy, documentation and data quality, and management support emerged as subthemes related to health care provider challenges, and are narrated as follows.

#### Subtheme 3.1: Training quality challenges

The findings of the study reflected and revealed that even though implementers were trained, implementation of the interventions they were trained in was not carried out. As a result, the quality of training was questioned, which is narrated in verbatim as follows:

‘You will find that people who have been trained on the PMTCT policy move from one section to another section of the facility, and sometimes you will find that you have trained certain institutions, and when you visit the institution, you find people not well versed with the policy.’ (P4, female, manager)

‘I am not sure whether they didn’t reach out to everyone, or the clinicians are not keeping themselves updated, or are forgetting, or they’re too lazy to open the policy just to ascertain exactly what they need do to manage the client. So, non-compliance has been observed, and it’s something that the programme would have to deal with.’ (P3, female, manager)

#### Subtheme 3.2: Guideline literacy

Regarding inadequate knowledge by clinicians, the implementers were categorised as being ‘ignorant, lacking motivation, not interrogating the guidelines, not adequately well versed, lacking understanding, not aware of the guidelines, not adequately trained’ based on the fact that, although they were trained, some had no interest in listening to what the coordinators were training them in. The verbatim responses are presented and discussed as follows:

‘You know, as clinicians, we ought to have our guidelines nearby so that when you are faced with a patient who is presenting in whatever way, you should be able to reach out to the guideline and check as to what the guideline says in terms of how to manage that and clinicians are no longer doing that. They are no longer consulting the guideline. We actually tried to make it easy by availing the guidelines on a WhatsApp group. So that one can just go through WhatsApp to check what is it that is required, but even though that strategy is like it’s not used.’ (P3, female, manager)

‘You will find that people who have been trained on the policy move from one section to another section, and sometimes you will find that you have trained certain institutions, and when you visit those institutions, you find people that are not well versed with the policy.’ (P8, female, manager)

#### Subtheme 3.3: Documentation and data quality

Data practices challenge the findings, which revealed poor data capturing and poor recording. This translated to missed data and information, reflecting the inaccurate recording of events in respective repositories, portraying incorrect performance and programme outcomes. Verbatim responses are narrated as follows:

‘So, during the ante-natal visit, what I found was that the viral load was done, but it was not necessarily captured in the system. At this specific facility, they’ve got FIKAs, and previously, we made use of Tier.net. In the delivery room, what I found was that the mothers’ viral load was not captured in the system. They were also not identified as pregnant women’s specimens. So, how the information has been captured in the different systems contributes to the 50% that the National Department of Health has detected regarding the province’s performance based on the information that I gathered. Poor capturing is part and parcel of this situation that we are finding ourselves in.’ (P9B, female, manager)

‘When the policy was implemented, the midwives didn’t know exactly how to capture the viral load, because concerning the tools, the department was still busy reviewing the data element. What I’m saying is that the review of the policy didn’t go in conjunction with the review of the data element. The data element review was lagging, so it created a problem for the midwives to capture.’ (P3, female, manager)

#### Subtheme 3.4: Health care provider challenges

The study’s findings and challenges, attributed to health care providers’ non-compliance in advocating for PMTCT interventions, included poor maternal viral load monitoring and poor PCR monitoring. Various factors were cited as rationales for non-performance or failure to perform interventions deemed critical to reducing the risk of MTCT. All the participants viewed non-adherence to interventions by implementers (clinicians) as a major contributor to the partial implementation of PMTCT policy and poor programme outcomes. The following are examples of the views shared by the participants:

‘“Poor maternal viral load [*VL*] monitoring” Midwives are reluctant to take blood in the delivery facility giving rationale that clients will be discharged by the time the results come back, and strongly felt that bloods for [*VL*] monitoring should be done at the post-natal facility the clients are seen post-delivery.’ (P1, female, manager)

‘“Poor polymerase chain reaction monitoring – 10-week PCR,” was seen as a missed opportunity as most babies are lost to follow-up, hence PCR not done according to advocated intervals.’ (P9, female, manager)

#### Subtheme 3.5: Management support

A lack of management support was viewed to be the cause of employees’ low morale, described as demotivating clinicians in implementing policies or demotivating them to do anything positive towards any work-related issues, which is described as follows:

‘Systemic issues are hardly corrected. You will find that staff morale is very low, and they may not do their work because they tell themselves, “I don’t care anymore, because I have been complaining, but nothing is happening.” On the other hand, the system is also to blame, because if we were looking after our employees’ well-being, if people are tired, there is nothing that can be done. After all, when they work, they just work to finish, they don’t have that “vooma” of doing the right thing.’ (P7, female, manager)

### Theme 4: Client-level determinants

The study findings reported various client-related challenges, such as antenatal care (ANC) late booking, clients defaulting on treatment (ART) and denial of HIV status by pregnant women, resulting in them being dishonest, stigma, babies lost to follow-up and a lack of knowledge by clients. The challenges were viewed by participants as hindering quality care and resulting in unwarranted negative outcomes. The impacts are highlighted and shared as follows.

#### Subtheme 4.1: Late antenatal care booking

The findings revealed that pregnant women are booking late for their ANC services, resulting in unwarranted consequences:

‘Our mothers do not book early, resulting in them receiving necessary intervention late in their pregnancies, some of them, having babies who sero-convert, because they either were initiated late on treatment; some of them, even when initiated on time, they do not adhere to their treatment regimens.’ (P8, female, manager)

‘We are still having cases where the mothers are reporting late to facilities for ANC booking, where some of them will not even come for ANC; they will just come for delivery. Then when they are tested, because their viral load is not known, the baby will be put on dual prophylaxes and then now when the results come, because the results do not come immediately, so when they come, they have to be taken to the facility where the referral facility is, because this baby has not attended to at the nearest facility, you find when the results come back maybe the viral load is suppressed, which means that the baby was not supposed to be on dual prophylaxes, because the results were not known, so it has to be stopped and continue with single prophylaxes, so there are those issues.’ (P1, female, manager)

#### Subtheme 4.2: Antiretroviral therapy adherence

Participants’ views were that when clients default on treatment, it leads to negative consequences, including an inability to achieve viral suppression, thereby increasing the risk of vertical transmission:

‘Others are not staying in the country. They will just cross over the border illegally to access treatment and go back home, and when they are supposed to come back in a month, they do not have money to bribe the people who are letting them in the country illegally. So, they will stay home without medication and miss the ANC visit until they get money, then you will see them again.’ (P6, female, manager)

‘You find that the client who was positive with the previous pregnancy, initiated on ART, took treatment maybe for a year and then after that, decided to stop, like the defaulters. If I may put it like that in the relevant term for ART, defaulted on treatment.’ (P1, female, manager)

#### Subtheme 4.3: Denial of human immunodeficiency virus status by pregnant women

The findings reported incidences of women shopping around facilities, hoping to be told a different story about their HIV-positive status. A sign of denial, coupled with dishonesty regarding where they reside, where they will be attending ANC services, as well as relocation without requesting a transfer letter, and giving wrong addresses, makes it difficult for the clinicians to render appropriate care:

‘Others move around the facilities within the same area. She will just be shopping around. I do not know; they say they are hoping to hear a different story, and whenever she presents in the facility, she is new, never tested before, then she’ll be tested. She’ll be booked and given treatment. Two weeks later, she goes to another facility. “I am new, I am pregnant, and I have come for a booking.” As for HIV medication given, they just do that thing. That is one thing that makes their management difficult sometimes. Others not staying here tend to go to where the husbands are working in mines to stay there.’ (P6, female, manager)

‘I have indicated that even our clients are not honest about where they are, like where they are going to deliver and where they are going to continue post-natal. At times, some clients are very much aware of their HIV status, but do not do the relevant follow-ups. Not coming to bring the child in for testing and for taking treatment, and we have clients who know they are HIV positive. They are on ART, but they still are not taking treatment. So, it is actually a multi-factorial problem which needs, you know, clients, clinicians, a functional system.’ (P10, female, manager)

#### Subtheme 4.4: Stigma

Stigma is one of the study findings experienced by the respective provinces, which is shared by participants as follows:

‘Men are not testing; they are testing through their female partners. If she comes back being positive, the man will take her tablets and say that they will give you more at the clinic, they don’t know me. So, let us share half-half. If yours are finished, you go and fetch more there.’ (P5, female, manager)

‘Tell them they got lost or you left them in the taxi or wherever. So, our males are not yet, I do not know what to say, they are still hiding, if I may put it like that. So, I can safely say that now in this instance, the women are not really, how can I say this, you know, when they used to say cover the tale.’ (P6, female, manager)

#### Subtheme 4.5: Lost to follow-up

A challenge of babies lost to follow-up and unaccounted for was highlighted:

‘The follow-up of the babies, once the baby is lost to follow-up in our province, we do not have a system to identify where the baby is … so you cannot sort of like trace them until the mother comes back.’ (P6, female, manager)

## Discussion

The findings of this study presented the experiences, views and challenges experienced by PMTCT managers during PMTCT policy implementation at service delivery platforms across SA. The study setting included all nine provinces in SA. The selection represents the views and perceptions of provincial PMTCT managers and the NDoH PMTCT from urban and rural areas. The findings showed partial implementation of the PMTCT policy, the implication signifying potential inability of the country to achieve eMTCT and have an AIDS-free generation by 2030.

All participants provided a rationale for their views on the partial implementation of the PMTCT policy, ranging from the design and change management (policy obstacles and frequent guideline change), system and service delivery constraints, and provider-level determinants and client-level determinants, to attributing the provider-level determinants as the most contributing factor.

An in-depth literature review was conducted. Furthermore, detailed implementation gaps, barriers and challenges were identified globally. These indicate only partial implementation of PMTCT policies and raise concerns regarding the efficacy of PMTCT policy and programme as a vehicle for achieving eMTCT and an AIDS-free generation by 2030. The findings from the study concur with the findings from previous studies conducted in SA.^23^

Inadequate human resources for health, because of staff attrition, was a major contributor to the partial implementation of the policy. A lack of staff motivation and communication was another factor that was identified. The findings of this study are consistent with those of prior studies.^10,19,24,25,26,27,28,29,30^

Coronavirus disease 2019 (COVID-19) lockdown regulations negatively impacted the PMTCT programme and policy implementation in SA, which is similar to the findings of other studies.^24,31,32,33^

A lack of management support and leadership involvement was viewed by the participants as the cause of employees’ low morale, thereby contributing to poor implementation of the PMTCT programme and policy, which is consistent with findings of other studies.^24,27,31,33^

Inadequate material resources related to the inconsistent provision of drug and HIV test kit stockouts were viewed as impediments for efficient PMTCT services, which is in agreement with the findings from previous similar studies conducted in SA.^19,31,34,35,36,37^

Poor programme management, overcrowding, reliance on partners and the lack of a sustainability plan by the government were blamed for the poor integration of services, which was viewed as missed opportunities to render mother and baby pair services, as well as non-functional two-way referral systems viewed as contributing to poor client management. These findings from the study concur with the findings of other previous studies.^10,19,26,27,31,37,38,39,40,41,42,43,44,45^

Health care provider challenges articulated by participants included non-compliance with PMTCT interventions, training challenges, poor record-keeping practices, and poor client management, all consistent with other study findings.^18,19,25,28,29,36,39,41,43,46,47,48,49^

Client-related challenges constituted by issues such as being late for ANC bookings, clients defaulting ART or failing to adhere to treatment, denial of HIV status by pregnant women resulting in dishonesty, babies lost to follow-up and a lack of knowledge by clients, were identified and verbalised by the participants and agree with findings from other studies conducted in SA.^10,13,19,25,26,27,28,29,32,28,33,36,43,48,50^

The stigma findings reported by participants to be prohibiting clients from accessing HIV and fuelling poor ART adherence concur with other research study findings.^27,35,36,43,48^

Guideline and policy changes are frequently reported as a major challenge in this study by participants, who have promoted the proliferation of numerous policies and protocols, causing a lot of confusion, negatively impacting the effective implementation of the PMTCT policy. This also concurs with other study results.^24,39,43^

### Limitations of the study

The study used purposive sampling, which recommended a sample size selection of approximately eight to 10 participants. The study purposefully selected provincial and national PMTCT managers’ perceptions.

Subdistrict and facility PMTCT managers were excluded. Their inclusion might have revealed different views and information emanating from the implementation level – the cold front, which might have been different from the perceptions, views and challenges experienced by the provincial and national PMTCT managers selected in this study.

## Conclusion and recommendations

The findings are organised into four interrelated thematic areas: policy design and change management, health system and service-delivery constraints, provider-level determinants and client-level determinants. The study’s findings provide important insights to inform future PMTCT policy development and implementation strategies. Addressing the identified challenges is essential to strengthening programme effectiveness and sustainability. In addition, sustained advocacy for PMTCT interventions and full, consistent implementation of PMTCT policies are critical to improving programme outcomes and supporting SA’s goal of eliminating MTCT of HIV by 2030. Implementing the guidelines proposed in Table 2 will also contribute to the elimination of MTCT by 2030.

**TABLE 2:** Guidelines to support the full implementation of the Prevention of Mother-to-Child Transmission policy across South Africa.

Findings	Proposed guidelines	Action point		
		Addressing policy design and change management	Addressing systemic challenges	Addressing provider-level determinants
**Policy design and change management**				
Policy obstacles and frequent guideline change	There should be a register and database for the approved policy changes.	The management support visit should establish whether facilities have the latest PMTCT policy.	-	-
Communication pathway gap	Communication from NDoH to disseminate provincial train-the-trainer sessions should be streamlined and structured.	Communication protocol for training respective cadres (train-the-trainer SOP) should be developed.	-	-
**System and service delivery constraints**				
Staffing levels – attrition, rotation of staff, and inadequate staffing	Personnel allocated to the PMTCT programme should be left to work for a continuous period of a year.	-	Adherence to the NDoH circular on non-rotation of mother and child programme implementers should be promoted.	-
Referral systems	A protocol should be developed for a two-way referral system between facilities and/or hospitals around provinces, inter-provincial and inter-country.	-	Communication of the two-way referral system using circulars and social media should be promoted and circulated.	-
Overcrowding	The department should apply the standardised clinician: patient consultation ratio applicable for rural and urban standards.	-	The appointment booking system for routine consultation and follow-up visits should be promoted and implemented.	-
Reliance on partners for PMTCT implementation	The DoH should increase dedicated funding for the PMTCT programme resources.	-	PMTCT managers should monitor if there is an increase and improvement in the budget allocation for the PMTCT programme.	-
Supply chain inadequacy	An SOP should be developed for the procurement and control of ARV drugs.	-	A bin card should be implemented to monitor the procurement and stock levels of ARV drugs.	-
Integration of services	The department must strengthen strategies to promote the integration of MCWH and sexual reproductive health services with other HIV care services as part of the PMTCT package.	-	Personnel should be re-orientated to integration strategies and marketing of MCWH and HIV care services.	-
Uncoordinated mother and baby pair service package	A one-stop service point should be reinforced to ensure that both mother and baby receive a comprehensive service package.	-	Clinicians should be re-orientated to understand the importance of a comprehensive care service provision for a mother and baby pair.	-
Lack of managerial support	There must be a system for recording management support visits to PMTCT sites.	-	A Management Support Visit Register should be kept at facilities rendering PMTCT services to record visits conducted.	-
Non-functional referral systems	A PMTCT referral system pathway must be included in the approved institutional pathway system.	-	An awareness programme for PMTCT referral pathway systems should be developed and marketed.	-
Reliance on partners for PMTCT implementation	The DoH must increase dedicated funding for the PMTCT programme resources.	-	PMTCT managers should monitor if there is an increase and improvement in the budget allocation for the PMTCT programme.	-
**Provider-level determinants**				
Non-adherence to PMTCT policy and protocols	There should be auditing of compliance with PMTCT policies and protocols.	-	-	Audit tools should be developed to measure adherence with the PMTCT policy and protocols.
Inadequate training of clinicians involved in the PMTCT programme	There should be a comprehensive PMTCT in-service training programme for PMTCT clinicians and/or guideline literacy.	-	-	PMTCT in-service training rendered by PMTCT managers should be implemented and monitored.
Inadequate knowledge by implementers	There should be a structured induction and orientation programme for all clinicians involved in PMTCT implementation.	-	-	A structured programme should be developed for the induction and orientation of clinicians involved in the PMTCT programme implementation.
Poor recording practices	Mandatory evidence submission of quarterly programme performance review.	-	-	It should be mandatory to submit evidence for the quarterly report submitted.
Clinicians not using available resources	A structured process should be developed to measure the utilisation of clinicians’ existing skills, knowledge, and competency.	-	-	An audit tool should be developed to measure the effective utilisation of skills and knowledge of clinicians at services rendering mother and pair care.
Lack of managerial support	There should be a system for recording management support visits to PMTCT sites.	-	-	A Management Support Visit Register should be kept at facilities rendering PMTCT services to record visits conducted.
**Client-level determinants**				
Clients defaulting on treatment	A support system must be developed to help clients adhere to treatment.	-	-	A PMTCT programme package should be developed to involve males and their partners to encourage and support their partners in understanding the importance of adherence to treatment.
ANC late booking	The PMTCT programme must be marketed adequately by using an alternative strategy.	-	-	PMTCT programme ambassadors should be appointed to market PMTCT services and raise community awareness.
Stigma	Alternative strategies must be developed to revitalise and relaunch PMTCT services, as well as to share information with communities.	-	-	The PMTCT programme should be relaunched to revive the programme to empower communities and share information for them to develop a sense of ownership.
Denial of HIV status by pregnant women and dishonesty	A strategy must be developed to support HIV pregnant women.	-	-	Community health care workers in respective areas should be allocated as a support system and buddies for pregnant women.
Babies lost to follow-up	A tracking system must be developed for HIV-exposed babies.	-	-	A health information tracking system needs to be implemented, maintained, and monitored to track clients’ movements in the entire country.
Clients lack knowledge	Alternative strategies must be developed to market PMTCT services and share information with communities.	-	-	National PMTCT campaigns should be marketed using community radio stations, television, newsletters, and billboards on community murals put up, as well as taxis branded.

PMTCT, Prevention of Mother-to-Child Transmission; NDoH, National Department of Health; SOP, Standard Operating Procedure; DoH, Department of Health; ARV, antiretroviral; MCWH, maternal, child, and women’s health; ANC, antenatal care; HIV, human immunodeficiency virus.
